# Nanomaterial-Mediated Electrochemical and Optical Biosensors and Their Application in Tumour Marker Detection

**DOI:** 10.3390/s25185902

**Published:** 2025-09-21

**Authors:** Xinlan Wang, Jingyi Hei, Tao Zhao, Xiyu Liu, Yong Huang

**Affiliations:** 1 State Key Laboratory of Targeting Oncology, Guangxi Medical University, Nanning 530021, Guangxi, China; wangxinlan@sr.gxmu.edu.cn (X.W.); heijingyi@sr.gxmu.edu.cn (J.H.); zhaotao@sr.gxmu.edu.cn (T.Z.); 2 National Center for International Research of Bio-Targeting Theranostics, Guangxi Medical University, Nanning 530021, Guangxi, China; 3 Guangxi Key Laboratory of Bio-Targeting Theranostics, Guangxi Medical University, Nanning 530021, Guangxi, China; 4 Collaborative Innovation Center for Targeting Tumor Diagnosis and Therapy, Guangxi Medical University, Nanning 530021, Guangxi, China; 5 Guangxi Talent Highland of Major New Drugs Innovation and Development, Guangxi Medical University, Nanning 530021, Guangxi, China

**Keywords:** nanomaterials, signal amplification, electrochemical biosensors, optical biosensors, tumor biomarkers

## Abstract

Cancer constitutes a category of diseases with high mortality rates, where early and precise detection plays a crucial role in diagnosis and treatment. Tumour markers are biomolecules produced during cancer progression, predominantly inert molecules that prove difficult to detect at low concentrations. Traditional detection methods, however, exhibit shortcomings in sensitivity and convenience. Biosensors, with their portability and high sensitivity, hold broad application prospects for detecting tumour markers. Nanomaterials, enhancing detection performance through signal amplification mechanisms, have increasingly become the primary choice for improving sensor analytical capabilities. This review retrieved 60 relevant publications from the Web of Science and PubMed databases (2018–2024) covering “nanomaterials, biosensors, tumour markers”, focusing on those employing signal amplification mechanisms and providing clinical sample validation. It summarises signal amplification mechanisms in nanomaterial-mediated electrochemical and optical biosensors, contrasting the differences between these two sensor types. This review focuses on the relationship between “nanomaterial functionality, signal amplification, and clinical application”. It systematises and presents the latest advances in nanomaterial-mediated biosensors for detecting tumour markers, analysing the challenges encountered in their clinical implementation. While providing guidance for the clinical translation of nanomaterial-mediated biosensors from laboratory research, their practical application still requires validation through further multicentre, large-scale studies.

## 1. Introduction

Cancer, as a major global health threat, continues to present a significant challenge to healthcare systems worldwide with rising incidence and mortality rates. Statistics indicate approximately 19.3 million new cases and nearly 10 million deaths in 2020 [1]. Cancer induces systemic disease through three primary mechanisms: rapid multi-organ metastasis, paraneoplastic syndromes, and cachexia. Considerable resources have been invested in cancer treatment, yet early diagnosis and detection remain pivotal to improving cure rates and survival outcomes. Effective early detection significantly reduces the risk of tumour metastasis and enhances patient prognosis, holding profound clinical significance [2,3]. Current diagnostic approaches commonly employed include routine physical examinations, invasive tissue biopsies, laboratory tests, PET-CT, CT, and MRI [4,5]. While routine physical examinations are relatively simple and non-invasive, they exhibit low sensitivity and necessitate supplementary diagnostic tools. Tissue biopsies can definitively determine the benign or malignant nature of lesions, yet they inevitably involve invasive procedures and sampling errors [6,7]. Laboratory testing reflects tumour marker expression levels but requires extended processing times, precluding point-of-care testing (POCT) [8]. Imaging techniques, while sensitive, entail high costs and significant radiation exposure, limiting their utility for diagnosing early-stage cancers. The development of precise and effective early-stage cancer diagnostic technologies is imperative.

Tumour markers are substances present in human body fluids and tissues, regarded as indicators for cancer diagnosis, progression assessment, and patient prognosis evaluation [9]. Numerous tumour markers are currently employed in clinical diagnostics, including proteins (such as alpha-fetoprotein (AFP) [10], phosphatidylinositol glycoprotein-3 (GPC-3) [11,12], prostate-specific antigen (PSA) [13,14], and carbohydrate antigens [15]), nucleic acids, and other biomolecules [16]. According to statistical data, liver cancer accounted for 8.3% of newly reported fatalities in 2020 [1]. Despite alpha-fetoprotein (AFP) serving as the gold standard for liver cancer diagnosis, 80% of small hepatocellular carcinomas and early-stage hepatocellular carcinomas remain undetected by AFP testing [17]. Nucleic acid biomarkers exhibit high sensitivity in cancer detection. Their combined use with low-dose computed tomography (LDCT) reduces the false-positive rate of LDCT from 19.4% to 3.7%. However, widespread adoption remains limited due to cost constraints [18]. PSA is the most commonly used marker for prostate cancer screening in men. Yet its overdiagnosis rate ranges from 17% to 50%, leading to unnecessary biopsies [19]. Traditional detection technologies such as chemiluminescent immunoassays, PCR, ELISA, and WB face challenges including high costs and low detection rates for low-concentration tumour markers, hindering early cancer diagnosis [20,21,22]. To address these issues and enhance detection performance, biosensors offer novel approaches to overcome the limitations of conventional techniques.

Biosensors are compact signalling devices capable of converting captured signals into visual representations. Based on the type of transducer employed, they can be categorised as electrochemical or optical biosensors [23,24]. Electrochemical biosensors, with their high accuracy, low fabrication costs, and potential for miniaturisation, have become the most widely used sensors. Researchers regard them as ideal devices for achieving point-of-care testing. Optical biosensors convert received signals into visually perceptible signals, offering a detection method with high specificity and sensitivity that holds broad application prospects. Table 1, compiled from reviewed literature, presents the advantages and disadvantages of electrochemical and optical biosensors.

To meet clinical diagnostic requirements, lower detection limits play a pivotal role in cancer diagnosis and patient prognosis. Sensor response signals require further enhancement, prompting the emergence of various signal amplification strategies, such as enzyme-catalysed amplification, nanomaterial-based amplification, and nucleic acid-based amplification. However, enzymes and nucleic acids are highly susceptible to external factors such as pH and temperature during reactions, necessitating optimisation of reaction conditions. Consequently, nanomaterial-based signal amplification strategies have gained greater favour among researchers. With dimensions ranging from 1 to 100 nm, nanomaterials exhibit distinctive properties owing to their small size and increased specific surface area. Nanomaterials enhance detection performance through the following mechanisms: ① high specific surface area (e.g., graphene) increases target binding sites via π-π bonds and electrostatic interactions, improving capture efficiency [25]; ② nanomaterials optimise the sensing interface to accelerate electron transfer and reduce reaction energy barriers [26]; and ③ enzyme-like activity, catalysing reactions through ionic valence changes with high efficiency and sustained activity. Surface modification through chemical or physical means not only preserves the inherent characteristics of nanomaterials but also enables diverse functionalities, achieving signal amplification in sensors and thereby enhancing detection sensitivity.

**Figure 1:**
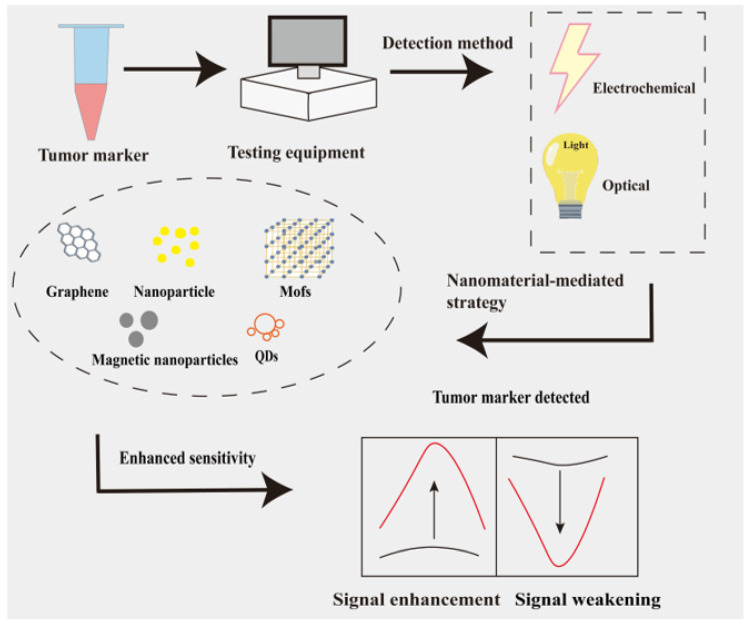
Schematic diagram of biosensors. Electrochemical and optical biosensors for detecting tumour markers. Mechanisms of action for different sensor types, with significantly enhanced sensitivity in nanomaterial-mediated sensors.

Existing reviews predominantly focus on single comparisons. Darwish et al. merely summarised the classification of nanomaterials in sensors without describing signal amplification mechanisms [27]. The Mousazadeh group concentrated on electrochemical biosensor applications without comparing them to other biosensor types [28]. Huang et al. solely reviewed single tumour marker (AFP) detection, failing to cover simultaneous multi-marker analysis [29]. The signal amplification mechanisms of nanomaterial-based sensors, alongside the application of nanomaterials in sensor construction and clinical translation, are equally pivotal (as illustrated in Figure 1). This review retrieved 501 publications from the Web of Science and PubMed databases covering the period 2018 to 2024, using the keyword combination ‘nanomaterials, biosensors, tumour markers’. Studies solely discussing nanomaterial synthesis without performance data were excluded, resulting in the inclusion of 60 representative publications. The review encompasses three key dimensions: electrochemical/optical sensors; metal- and carbon-based and MOF-type nanomaterials; and protein, nucleic acid, and exosome biomarkers. This review centres on signal amplification mechanisms in electrochemical and optical biosensors mediated by nanomaterials, detailing recent advances in enhancing sensing performance through nanomaterials. It compares electrochemical and optical biosensors, discusses pathways from laboratory research to translational applications, and supplements the application prospects of emerging antibody nanomaterials. This work provides reference for advancing the clinical application of nanosensors in cancer diagnostics.

**Table 1:** Advantages and Disadvantages of Electrochemical and Optical Biosensors.

Classification	Advantages	Disadvantages	Ref.
Electrochemical Biosensor	High sensitivity, rapid detection, low fabrication cost, and ease of analysis	Weak stability	[30]
Optical Biosensor	Strong selectivity, analytical accuracy, and ease of transport	Light quenching readily occurs, affecting detection results	[31]

## 2. Nanomaterials and Electrochemical Biosensors

**Figure 2:**
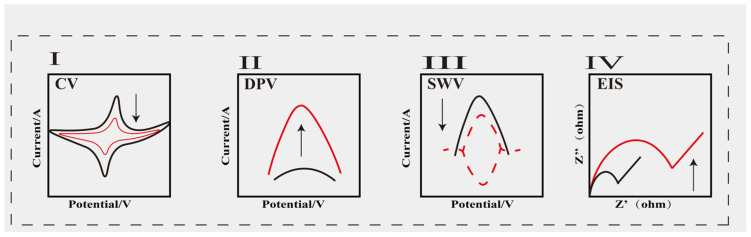
Variation diagram of four electrochemical detection methods: (**I**) cyclic voltammetry, (**II**) differential pulse voltammetry, (**III**) square wave voltammetry, and (**IV**) electrochemical impedance spectroscopy.

The operating principle of electrochemical biosensors involves a recognition element on the electrode undergoing a chemical reaction with the target substance, thereby inducing alterations in electrical signals. The electrical signals generated by the sensor correlate with the concentration of the target substance. Qualitative or quantitative analysis of the target substance is achieved by analysing either the current changes induced by the binding of the target substance to the electrode surface, or the potential response arising from redox reactions on the electrode surface [32]. Based on alterations in the electrical signal, researchers commonly employ cyclic voltammetry (CV), differential pulse voltammetry (DPV), electrochemical impedance spectroscopy (EIS), and square wave voltammetry (SWV) for detection [33,34,35]. Figure 2 illustrates the response signal variations obtained through four detection methods. These detection methods enable the evaluation of an analyte’s conductivity within the electrode system, the detection of current changes following redox reactions in electroactive substances, the observation of resistance alterations after charge transfer, and the measurement of potential differences between electrodes. To capture more satisfactory electrochemical signals, researchers incorporate nanomaterials into sensor construction, primarily amplifying electrical signals through three approaches: leveraging the catalytic properties of nanoenzymes, optimising the sensing interface with nanomaterials, and exploiting the structural advantages of nanomaterials. Within these three domains, approaches encompass analysing target substance concentrations through interface responses triggered by nanomaterial signal probes, as well as directly modifying electrode surfaces to enhance electrochemical response signals for target substance analysis. This chapter will address each method sequentially, presenting our insights.

### 2.1. Nanozyme-Mimetic Catalysis Amplifies Electrochemical Signals

Employing enzyme-labelled electroactive substances as signal probes to catalyse electrochemical reactions constitutes a common strategy for signal amplification. Enzymes are immobilised onto the sensor surface, utilising their catalytic activity to catalyse corresponding substrates, thereby amplifying the detection signal. During the early stages of biosensor development, researchers often prioritise enzyme-catalysed reactions due to their distinct advantages: exceptional specificity, high sensitivity, and mild reaction conditions. Horseradish peroxidase and alkaline phosphatase are the most frequently employed natural enzymes. For instance, Zhou’s team designed a dual-amplification electrochemical biosensing platform utilising HRP to catalyse the reduction of H_2_O_2_ in solution to O_2_, thereby amplifying the electrochemical signal for target analysis [36]. Lu et al. constructed an electrochemical biosensing platform for sensitive detection of miRNA-21 by integrating double-stranded specific nuclease (DSN) with three-dimensional DNA tetrahedral structure probes (TSPs), harnessing the electrocatalytic activity of enzymes [37].

Furthermore, variations in temperature and pH during sensor construction may reduce the catalytic activity of natural enzymes, thereby compromising the accuracy of detection [38,39]. To address this issue, researchers have developed nanoenzymes as alternatives to natural enzymes, drawing upon the principles of enzyme catalysis. Compared to natural enzymes, nanoenzymes are simpler to prepare and store. Furthermore, their catalytic activity can be modulated by altering their size and structure, representing a successful integration of nanotechnology and enzymology [40,41,42]. Wu’s team developed a two-dimensional MnO_2_ nanosheet capable of converting dissolved oxygen into reactive oxygen species, exhibiting both oxidase and peroxidase-like activities [43]. Upon binding with substrates, the DPV response signal remained stable. In the presence of the target miRNA let-7a, it forms strong hydrogen bonds with the nanosheets, shielding the catalytic sites and hindering substrate binding. This reduces enzyme-like activity, leading to a distinct change in the electrical signal. Under optimal reaction conditions, the detection range spans 0.4–140 nM, with a detection limit of 0.25 nM. Further structural modifications to the nanocatalyst enhance detection sensitivity. Li et al. developed an electrochemical sensor for miRNA-21 detection based on an MOF@Pt@MOF nanocatalyst and a cascade primer exchange reaction (PER) [44]. In the presence of the target, PER generates a long single-stranded DNA that exposes capture probes, enabling nanocatalyst binding to the sensor surface. The sandwich nanomaterial catalyses peroxidase activity, decomposing H_2_O_2_ to significantly amplify electrochemical signals. This sensor not only achieved a detection limit of 0.29 fM but also distinguished homogenous microRNAs with single-base mismatches. Fang employed Au@ZnO with peroxidase-like activity, concurrently labelling antibodies with HRP. This synergistic approach enabled sensitive detection of AFP [45].

Beyond directly catalysing electrochemical reactions, another pathway for enzyme catalysis to amplify response signals involves promoting the deposition of insoluble substances onto electrode surfaces. This enhances detection sensitivity by altering the current before and after deposition. Inspired by electrocatalytic precipitation reactions, nanoenzymes can catalyse the deposition of electroactive substances at the sensing interface to achieve signal amplification. Consequently, silver deposition and tyramine signal amplification techniques have been proposed [46,47]. Li et al. combined H-rGO-Pd nanoparticles with GPC3 aptamers to form labelled probes, modifying electrodes with Au NPs@rGO and immobilising GPC3 antibodies. In the presence of GPC3, both components form a sandwich complex with GPC3. The nanoparticle enhances the action of H_2_O_2_, enabling the in situ deposition of Ag^+^ from the AgNO_3_ solution onto the electrode surface. This significantly amplifies the DPV signal, thereby improving detection sensitivity [48]. Xie and colleagues first modified the sensing interface. Under the action of reduced graphene oxide-haem (RGO-haem) nano-enzymes, the target substance undergoes hydrolysis to produce H_2_O_2_, which then catalyses the substantial deposition of the electroactive substance ferrocene tyramine (Fc-Tyr) on the sensor surface, enhancing the response signal [49].

Different nanozymes exhibit distinct material compositions and structures, resulting in varying physicochemical properties. MnO_2_ nanosheets catalyse through metal ion oxidation–reduction cycles, whereas HRP relies on electron transfer from haem Fe^3+^, with optimal pH being neutral or weakly acidic. Catalytic efficiency of nanosheets diminishes under acidic conditions, necessitating pH adaptation optimisation for acidic tumour microenvironments. In practical applications, it remains necessary to regulate the active sites of nanoenzymes [50]. Furthermore, single-atom catalysts maximise atomic utilisation and accelerate electrocatalytic reactions through atomic-level active site regulation. Researchers modified individual platinum atoms on carbon nitride nanorods to prepare SA–Pt/g–C_3_N_4_–K composites exhibiting peroxidase-like activity. Enzymatic activity peaked at pH = 4 and 48 °C, demonstrating superior stability to HRP and higher POD-like activity, thereby enhancing detection sensitivity [51]. However, current research reports remain limited, necessitating further data to evaluate its biocompatibility and stability.

### 2.2. Nanomaterials Optimise Electrode Interfaces to Enhance Electrochemical Reactions

In electrochemical sensors, modifications to the electrode surface or electrode material through physical or chemical methods can alter electrical signals. These modifications may serve as switching elements for immobilising antibodies, antigens, or target proteins, thereby enhancing electrocatalytic activity, promoting electron transfer efficiency, and improving detection sensitivity. The concentration of the target substance and the electron transfer rate at the electrode surface are key factors influencing the electrochemical response signal. Modifying electrode surfaces with metal–nonmetal nanocomposites [52,53] or noble metal nanoparticles [54] not only enables the formation of stable structures with substrates but also enhances sensor stability and sensitivity.

**Figure 3:**
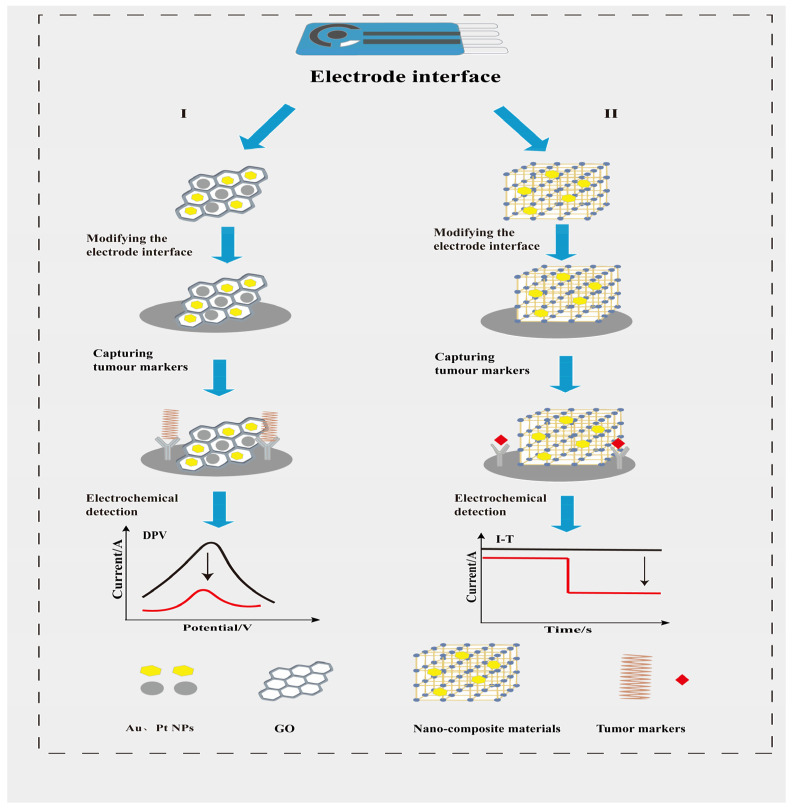
Schematic of optimised electrode interfaces with nanomaterials enhancing electrochemical reactions. (**I**): Optimisation of electrode surfaces using two types of nanomaterials that simultaneously enhance conductivity and surface area, such as gold and platinum nanoparticles combined with graphene nanomaterials. Following capture of tumour markers, DPV detection demonstrates increased sensitivity. (**II**): Optimisation of electrode surfaces using nanocomposites exhibiting electrocatalytic activity. Following capture of tumour markers, addition of hydrogen peroxide substrate yields enhanced sensitivity in I-T method detection.

Yan’s team employed a multi-step synthesis to prepare an electrocatalytically active manganese dioxide/hollow nanobox metal–organic framework (HNM) gold–platinum–palladium (AuPtPd) nanocomposite [55]. Following immunoreaction at the sensing interface, corresponding substrates were introduced for amperometric (I-T) detection of the tumour marker CA72-4. This approach not only minimised background current interference and significantly enhanced detection sensitivity but also achieved recovery rates for clinical samples ranging from 99.38% to 100.52%. Bharti enhanced conductivity by coating carboxylated graphene oxide onto fluorinated tin oxide (FTO) sheets [56]. Graphene oxide is readily modifiable; carboxyl-modified graphene oxide permits incorporation of numerous biomolecules onto its surface while providing binding sites for AuPt NP deposition [57]. The capture probe was immobilised on the electrode surface utilising the biotin–streptavidin interaction. In the presence of miRNA 21, the DPV response current exhibited a significant change. This strategy, which employs electrocatalytically enhanced electrochemical reactions without requiring specific enzyme catalysis, plays a crucial role in enhancing sensor sensitivity. Design strategies for improving sensor sensitivity using different substrate nanomaterials are illustrated in Figure 3.

Researchers have combined quantum dots with nanomaterials to form composite materials, integrating the reactivity of quantum dots with the conductivity of nanomaterials. This synergistic approach enhances the electrochemical performance at the electrode interface, enabling the detection of multiple tumour markers, particularly nucleic acid molecules. Maryam et al. prepared gold nanoparticles and CdSe@CdS quantum dots as nanocomposites for use as sensor markers. They then modified the electrode surface with polythiophene and reduced graphene oxide, enhancing both surface conductivity and π-π conjugation capabilities [58]. The nanocomposite underwent complementary hybridisation with capture probes on the electrode surface. Utilising DPV to record signals from both composites enabled simultaneous detection of two tumour markers. In another report, Pothipor constructed a sensing platform capable of concurrently detecting three breast cancer-associated miRNAs [59]. The electrode was modified with gold nanoparticles/graphene quantum dots/graphene oxide (AuNPs/GQDs/GO), providing a large specific surface area and strong electron transfer capability. Capture probes for the three miRNAs were immobilised on the electrode surface. Following hybridisation with targets, the SWV peak current of the redox probe decreased significantly, enabling sensitive detection of miRNA-21, miRNA-155, and miRNA-210. This sensing strategy exhibits high selectivity towards mismatched sequences, coupled with favourable repeatability and stability. The rapid and accurate simultaneous detection of multiple tumour markers not only conserves samples and time but also enhances diagnostic efficiency, meeting clinical demands for high-throughput testing.

Conventional antibodies possess large molecular weights and frequently encounter issues such as high-temperature denaturation during sample processing and interference from acidic conditions within the tumour microenvironment, limiting sensor applications under complex conditions. Variable regions of heavy-chain antibodies (VHH), variable regions of neoantigen receptors (VNARs), and variable lymphocyte receptors (VLRs) serve as novel small-molecule antibody platforms. Leveraging their unique structural stability and functional properties, these can combine with nanomaterials to form ‘carrier-recognition units’. VHH represents the variable region fragment of camelid ‘heavy-chain antibodies’ naturally lacking light chains, comprising a single polypeptide chain. Its core structure consists of three complementary determining regions (CDRs) responsible for antigen binding and four framework regions (FRs) maintaining structural stability [60]. Sadaf et al. employed porous gold electrodes as substrates, immobilising anti-VEGF VHH antibodies onto the electrode surface to construct an electrochemical immunosensor for VEGF detection [61]. This sensor exhibited a linear range between 0.1 pg/mL and 0.1 µg/mL, with a detection limit of 0.05 pg/mL. Research indicates that VHH’s CDR regions form a ‘convex ring’ structure, enabling tight binding to antigenic concave epitopes. Under optimal storage conditions, it exhibits excellent long-term stability. Even after 48 h in a protease-containing environment, VHH retains over 90% of its binding activity, demonstrating superiority over traditional IgG. This characteristic offers novel insights for developing in situ detection of CEA in gastric fluid from gastric cancer patients. VNARs possess a smaller molecular weight, comprising two constant regions and one variable region. Lacking light chains and featuring conserved cysteine residues within the framework regions (FR2, FR4), they exhibit tolerance to high salt concentrations and denaturing agents. Leveraging this property, Gao and colleagues isolated a VNAR targeting Helicobacter pylori adhesin A from an immunised shark VNAR library. They constructed a sandwich ELISA for detecting this adhesin [62]. The anti-HpaA VNAR and bivalent VNAR retained binding activity after incubation for one hour under acidic conditions at pH 1.2, resolving the issue of traditional antibody inactivation in strongly acidic gastrointestinal detection. The constructed ELISA achieved a detection limit down to the nanogram level, making it suitable for highly sensitive disease diagnosis. VLRs originate from agnathans and differ structurally from the other two types. They comprise 20–30 leucine-rich repeat (LRR) sequences forming a horseshoe conformation, achieving antigen-binding diversity through LRR module recombination [63]. The antigen-binding domain of VLRs is generated through autonomous recombination of LRR variable regions, enabling recognition of conserved epitopes that are difficult for conventional antibodies to bind (such as bacterial cell wall polysaccharides). VLRs also exhibit tolerance to high temperatures and acidic environments, making them suitable for sensing and detection applications in complex conditions.

Nanomaterials enhance electrochemical reactions by optimising electrode interfaces to improve electron transfer efficiency, offering a relatively straightforward operational process. However, most sensors rely on electrode undercoating deposition methods, making the stability of nanostructures on electrode surfaces a critical issue requiring urgent research and improvement. The detection limits of AuPtPd nanocomposites vary between batches, necessitating further optimisation of the synthesis process to enhance the reproducibility of the sensors. In future research, scientists may improve interface stability by refining self-assembly techniques (such as optimising Au-S bonds), employing multi-step electrodeposition to modulate the bonding strength between nanomaterials and electrodes, or developing novel nanobodies [64].

### 2.3. High-Surface-Area Nanomaterials Enhance Electrocatalytic Activity

**Figure 4:**
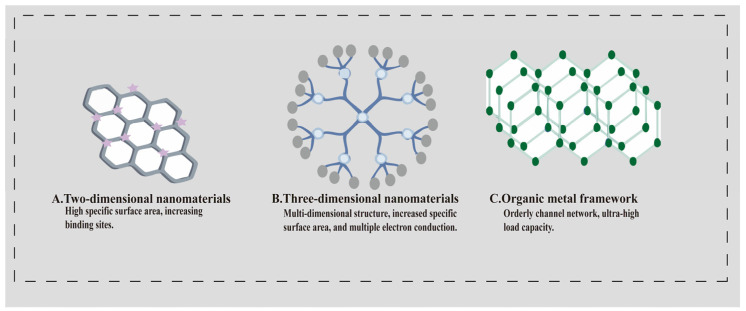
Schematic illustration of the mechanism by which high-specific-surface-area nanomaterials enhance electrocatalytic performance. (**A**). Two-dimensional nanomaterial graphene: Provides high specific surface area through a honeycomb planar structure; asterisks denote π-π bond adsorption sites capable of immobilising biomolecules. (**B**). Three-dimensional nanonetwork: Scattered branching increases specific surface area relative to two-dimensional materials; grey dots indicate multi-point electron conduction pathways. (**C**). Metal–organic frameworks: Ordered hexagonal pore networks enable ultra-high electroactive molecular loading.

In the construction of electrochemical biosensors, the high specific surface area of nanomaterials enhances the loading capacity of target molecules and increases the number of electrocatalytic active sites [65]. Such structural benefits are typically manifested in two-dimensional, three-dimensional, and organometallic framework materials, as illustrated in Figure 4.

To increase the specific surface area of electrodes and enhance biomolecular enrichment, certain nanomaterials possessing both large surface areas and excellent conductivity have been introduced, such as carbon-based materials. Carbon-based materials have found extensive applications in environmental sanitation testing, tumour marker detection, and targeted drug delivery [66]. Graphene, the most prevalent carbon-based material, achieves efficient loading of target molecules through physical adsorption via π-π bonds with biomolecules. To meet the clinical demand for highly sensitive detection, the inherent physical adsorption stability of graphene alone proves limited. Researchers have optimised its performance through composite or doping modifications to enhance utilisation efficiency. Mohamed designed a sensor based on pyrocatechol (PCA)-functionalised reduced graphene oxide (rGO) [67]. This sensor employs terminal amino groups to bind DNA capture probes, whilst AuNPs are modified with 6-ferrocene-2-thiol (Fc-SH) to generate an oxidation peak during electrochemical reactions. In the presence of the target, the capture probe forms double-stranded complexes with the target on the electrode surface, hindering electron transfer and reducing the Fc oxidation peak current. This sensor not only lowers the detection limit for miRNA-21 but also achieves an overall reaction time of just 30 min, outperforming conventional detection methods. Yu et al. designed a biosensing platform based on nanoparticle-doped N-reduced graphene oxide (N-rGO) through doping modification [68]. PtNi nanoparticles are uniformly distributed across N-rGO, with nitrogen atoms grafted onto the graphene surface via the graphitic nitrogen structure, anchoring additional PtNi nanoparticles through a physical adsorption process. Simultaneous dual-channel redox reactions in the electrolyte, combined with the synergistic effect of metal nanoparticles, significantly enhanced the electrochemical response signals at the electrode surface. Consequently, carbon-based materials can maximise signal amplification by strategically regulating their structural properties to optimise specific surface area utilisation.

Two-dimensional nanomaterials offer substantial specific surface area within a planar plane, yet spatial utilisation remains constrained. Three-dimensional nanomaterials featuring porous structures or branching configurations address this limitation by facilitating contact between reactants and active sites through pore networks or branching pathways. This enables dual-channel redox reactions, thereby enhancing electrocatalytic performance [69]. The Chen team utilised polyethylene glycol to transform PdAuCu nanocrystals into three-dimensional sea urchin-like nanocrystals, forming a nanocomposite with ferricyanide-grafted polylysine (Fc-g-PLL) as a signal probe [70]. Upon CEA capture onto the electrode surface, the signal probe binds to it, catalysing the amplification of current signals. This three-dimensional structural design can be tailored to create specific shapes as required, thereby reducing the amount of biomaterial used. It can even be employed in the construction of wearable sensors, designed to conform to the wearer’s body according to the intended wearing location.

In sandwich-type electrochemical biosensors, combining high-surface-area nanomaterials with signal probe loading achieves signal amplification through the ‘marker–target–probe’ triad. Metal–organic framework materials have emerged as the most ideal carriers. Chang utilised porous UIO-66-NH_2_ as a nano-framework, capable of encapsulating two electroactive dyes (MB and TMB) separately. Complementary double-stranded DNA targeting the dyes was bound to the metal framework surface, acting as a ‘guardian’ [71]. The DNA strands on the MOF surface inhibit dye release, resulting in extremely low peak currents for MB and TMB. Upon the presence of target miRNAs let-7a and miRNA-21, DNA strands on the MOFs form RNA-DNA complexes via complementary base pairing, subsequently detaching to release encapsulated electroactive dyes and generate strong peak currents. This structural design achieves multiplicative amplification of the response signal.

High-surface-area nanomaterials achieve signal amplification through a dual mechanism: (I) increasing the loading sites for target molecules to enhance reaction concentration; (II) providing more electrocatalytically active centres via porous, branched, and other structures to accelerate electron transfer. From optimising the sensing interface of two-dimensional graphene to the provision of multiple electrocatalytically active sites through three-dimensional materials’ branching structures, and further to the carrier function of metal frameworks, structural design of nanomaterials continues to advance, establishing itself as a core strategy for signal amplification in electrochemical biosensors. By regulating the dimensions, pore sizes, and surface modifications of materials in future, the utilisation efficiency of nanomaterials can be further enhanced, providing a more effective structural foundation for ultra-high-sensitivity detection.

## 3. Nanomaterials and Optical Biosensors

Optical biosensors, owing to their exceptional detection capabilities, find applications across diverse fields including medicine, biological research, the food and pharmaceutical industries, and environmental monitoring [72]. During the detection process, specialised biodetection components are employed. When the target substance reacts chemically or biochemically with the probe, this induces alterations in light intensity. Monitoring devices capture the optical signals generated during this reaction, constituting the fundamental principle of optical biosensors [73]. Compared to conventional detection techniques, optical biosensors constructed using nanomaterials exhibit numerous advantages, such as compact size, rapid response, real-time monitoring capability, and considerable cost-effectiveness. These sensors leverage principles of plasmonics, photonics, and the enhancement effects of nanomaterials to convert biomolecular interactions into detectable optical signals. They can detect analytes at extremely low concentrations, such as attomoles (aM) [74]. Depending on the optical transducer employed, these can be categorised as: surface-enhanced Raman spectroscopy (SERS) [75], colourimetric, localised surface plasmon resonance (LSPR) [76], electrochemiluminescence [77], and fluorescence sensors [78].

### 3.1. Surface-Enhanced Raman Scattering (SERS)-Based Nanosensor

Surface-enhanced Raman scattering (SERS), first observed in 1974, constitutes a highly efficient vibrational spectroscopy technique. Compared to other optical detection methods, SERS-based optical sensors exhibit distinct characteristics, enabling the assessment of structural information associated with novel compounds and providing novel insights into unknown substances, thereby functioning akin to a ‘fingerprint’ [79,80,81]. Researchers have discovered that introducing different nanoparticles enables accurate and sensitive detection of analytes, amplifying the acquired Raman signals [82].

Leveraging the unique properties of nanomaterials, scientists have proposed a dual-mode strategy integrating diagnosis and treatment. Ovarian cancer remains the most prevalent cancer among women today, presenting with subtle clinical symptoms in its early stages that are difficult to detect, often leading to advanced-stage diagnosis [83]. SERS serves as an efficient method for measuring extremely low concentrations of substances. Lan and colleagues employed SiO_2_ as a coating material to load gold nanostars@gold–silver core–shell nanostructures and SERS for evaluating cyclophilin A (CYPA), a biomarker associated with ovarian cancer [84]. Au@AgAu YSNS was constructed through a stepwise process, subsequently coated with SiO_2_, and finally conjugated with antibodies. The Au-Ag nanomaterial exhibits enhanced SERS performance, achieving a detection limit of 7.76 × 10^−10^ μg/mL. However, in haemolysed samples, SERS signals fluctuate, affecting detection outcomes. Additionally, this nanomaterial possesses outstanding photothermal properties, enabling its use as a photothermal agent for photothermal therapy, also termed a ‘nanodiagnostic-therapeutic agent’. Another study proposed a dual-mode fluorescence-SERS biosensor for detecting Mucin1, a transmembrane protein overexpressed in tumour cells [85]. DNA complementary to MUC1 was modified onto gold nanoparticles, forming competitive binding with aptamers modified by quantum dots. Precise protein quantification and imaging were achieved by quenching the quantum dot fluorescence signal and significantly enhancing the SERS signal through the metal fluorescence quenching effect and the hot-spot effect of the gold nanoparticles. The flexible application of nanomaterials and the innovative proposal of dual-mode sensing technology provide novel approaches for cancer diagnosis.

Chen et al. employed a surface-enhanced Raman scattering (SERS) sensing method utilising graphene oxide nanorolls encapsulating AuNPs to detect dozens of amino acids in saliva, distinguishing between gastric cancer patients and healthy individuals during morning and evening periods [86]. Graphene oxide was selected as the substrate, with graphene oxide nanorolls encapsulating gold nanoparticles subsequently prepared via an ultrasonication method. A/GO NSS adsorbed biomarkers and amplified their Raman signals, enhancing detection sensitivity. To validate the sensing performance, 220 clinical samples were analysed, achieving 80% sensitivity in successfully distinguishing gastric cancer patients from healthy individuals during morning and evening periods.

To meet detection requirements, researchers began reacting nanomaterials with target substances before structural modification. Pan and colleagues developed novel nanoprobes utilising gold nanostar-modified molybdenum disulphide nanosheets (MoS_2_-Au NSs) for detecting CD63 associated with gastric cancer [87]. Rhodamine X (ROX) was labelled onto aptamers recognising CD63 (ROX-Apt) and immobilised onto the nanosheet surface. The presence of ROX enhances the SERS signal. When exosomes are present, the formation of ROX-Apt–exosome complexes leads to signal attenuation, providing a valuable reference for early cancer diagnosis.

SERS performance is influenced by multiple factors, such as the type of chemical bonds between nanoparticles and molecules, the wavelength of light excitation, and the surface characteristics of nanoparticles. To address these challenges, researchers may adopt the following approaches: Firstly, characterising the morphology of nanomaterials and precisely controlling the interparticle spacing can enhance material properties. Secondly, focusing on surface functionalisation of nanomaterials—selecting specific aptamers and employing diverse functional groups for modification—enables adaptation to broader excitation wavelength ranges. This facilitates the development of more sensitive optical sensors for detecting tumour markers.

### 3.2. Nanoscale Sensors Based on Colourimetry and Localised Surface Plasmon Resonance (LSPR)

Given the complexity and diversity of cancer, traditional detection methods alone prove inadequate, as certain tumour markers at extremely low concentrations in early stages are difficult to detect. Consequently, more sensitive detection methods are required. Researchers employ colourimetric assays to compare colour changes induced by chemical reactions between target molecules and probes. Following binding between the target and the colourimetric probe, light absorption occurs within the visible spectrum. Researchers can thus obtain results without requiring expensive detection equipment [88]. Colourimetric methods, characterised by their simplicity and low cost, have garnered significant attention from researchers. In recent years, the introduction of various nanoparticles has enhanced the detection performance of colourimetric methods, amplifying detection signals. This holds promise as a prospective technology for detecting actual biological samples.

Li et al. developed a nanocatalyst-based colorimetric sensing system capable of detecting and distinguishing three common biological thiols (Cys, Hcy, and GSH) [89]. First, a Ag-MoS_2_ hetero-nanocatalyst was prepared via an in situ self-reduction reaction between MoS_2_ and Ag^+^. This nanomaterial acts as a highly efficient catalyst, decomposing H_2_O_2_ to convert the colourless chromogenic substrate tetramethylbenzidine (TMB) into a blue colour. However, the colourimetric reaction is pH-dependent. The -SH groups in biological thiols form metal-S bonds with the metallic nanophase, reducing the electron density of the nanomaterial and thereby inhibiting catalytic activity. At different pH levels, the binding behaviour between the Ag and Mo metal centres and the –SH groups varies, resulting in distinct colour changes. Researchers employed TMB–H_2_O_2_ colourimetric reactions under three distinct pH conditions, which produced different ‘reaction traces’ for these three biological thiols. Detection was achieved through compositional analysis. The detection limits for the three biological thiols were 0.72 μM, 0.89 μM, and 3.28 μM, respectively, successfully distinguishing differences between hepatocellular carcinoma cells and normal cells.

**Figure 5:**
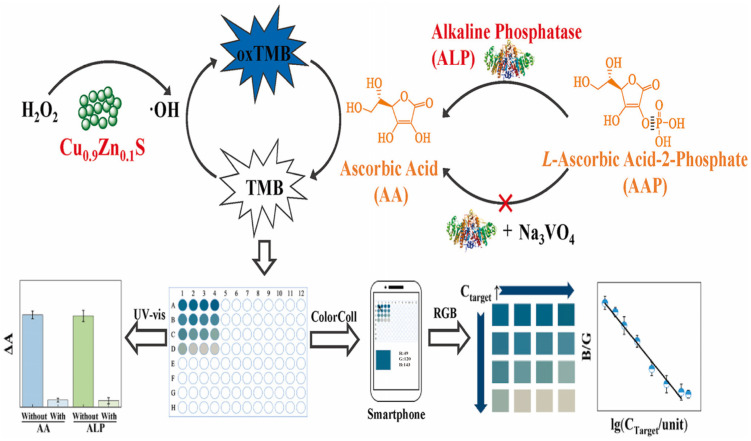
The schematic diagram illustrates the detection process of a colourimetric reaction catalysed by nanoenzymes. Reprinted from [91]. Copyright (2024), with permission from Elsevier.

Alkaline phosphatase (ALP) is recognised as a biomarker for conditions such as hepatic dysfunction and cancer [90]. Given the growing clinical demand for diagnostic tools that combine precision with portability, Cheng and colleagues developed a method for quantifying ALP within cells using the imaging capabilities of a smartphone [91]. The mechanism of this colorimetric sensor is illustrated in Figure 5. Through a simple doping method, Zn(II) ions were incorporated into CuS to yield Cu_0.9_Zn_0.1_S nanoparticles. Enzyme-active nanoparticles, upon encountering hydrogen peroxide (H_2_O_2_), can catalyse the conversion of TMB, resulting in a visible colour change in the solution. Subsequent addition of ascorbic acid (AA) reduces the oxidised TMB, causing the colour to fade. If cells contain alkaline phosphatase (ALP), L-ascorbic acid-2-phosphate is enzymatically converted to AA, which reduces TMB to colourless. A smartphone captures the colour change, and the ALP content is assessed by measuring the RGB values of the image. This sensor exhibits a detection limit as low as 0.47 mU/L and demonstrates excellent repeatability.

Metals or metallic nanomaterials exhibit a unique property known as localised surface plasmon resonance (LSPR). This phenomenon occurs when the frequency of incident light resonates with the collective oscillation of free electrons [92,93]. As a prevalent optical sensing technique, LSPR has been employed by researchers for highly sensitive real-time detection.

Precise control over the size, shape, and surface properties of nanomaterials can optimise the LSPR effect, enhancing the sensor’s detection performance for tumour markers. Trastuzumab (HER2), a specific therapeutic agent for HER2-positive breast cancer, was selected as the detection target. Shahbazi et al. devised a sensing strategy based on the interaction between trastuzumab (HER2) and nanoparticles, comprising two modules: signal generation and capture [94]. The signal module primarily comprises Au NPs and Ag NPs, modified with citrate ion-capturing groups and mixed, which interact with HER molecules. The two modified nanoparticles are prepared, mixed in a specific ratio, and adjusted to the target pH. The negatively charged citrate ions in the sensing structure selectively bind to the positively charged amino groups of the HER molecule, promoting hydrophobic interactions between the HER hydrophobic pocket and the Au NPs. This interaction alters the medium surrounding the nanoparticles, inducing changes in the LSPR spectrum. HER concentration is quantified via absorbance ratio, with the sensor exhibiting a linear range of 0.5–40 × 10^−7^ M. In another report, Meng and colleagues employed an LSPR-enhanced Ag@Au nanostructure biosensor for detecting miRNA let-7a [95]. The team first synthesised luminol–gold nanoparticles and then employed palladium nanoclusters as catalysts with hydrogen peroxide as the co-reactant. The Ag@Au nanostructure served as the LSPR source, enhancing the sensor’s sensitivity by amplifying the initial anodic signal. Following chain displacement amplification, the target could be converted into output DNA for detection. This sensor exhibits a detection limit of 5.45 aM.

Nanomaterial-mediated LSPR technology demonstrates outstanding sensing performance, yet practical application remains challenging. For instance, silver nanoparticles—the core material in LSPR technology—undergo oxidation reactions in serum, leading to diminished LSPR signals. Prolonged use may also induce nanoparticle agglomeration, further compromising sensor stability. Consequently, researchers may consider adopting ‘shell–core’ structures such as SiO_2_@Ag during nanomaterial preparation to enhance stability and mitigate the chemical reactivity inherent in metallic nanomaterials. Concurrently, anti-fouling coatings should be applied to prevent non-specific adsorption. Developing novel corrosion-resistant nanomaterials for substitution represents another avenue. These approaches collectively advance the practical application of LSPR technology.

### 3.3. Nanosensor Based on Electrochemical Luminescence

The signal generation process in electrochemiluminescence (ECL) sensors involves substances on the electrode surface being subjected to voltage, undergoing high-energy electron transfer to produce excited states, and ultimately emitting light signals [96]. This method combines the advantages of two technologies, possessing both optical sensitivity and a broad linear range alongside the simplicity and stability characteristic of electrochemical techniques. ECL sensors do not rely on excitation light and are unaffected by background signal interference, an advantage particularly evident in sensing detection and imaging applications. An increasing number of ECL sensing strategies have been proposed, with laboratories applying them to health analysis for cancer patients [97]. In constructing ECL sensors, commonly used luminescent reagents are primarily categorised into three types: organic small molecules, inorganic compounds, and nanomaterials. Among these, ruthenium(II) tris(bipyridine) (Ru(bpy)_3_^2+^) and luminol represent two highly representative electrochemical luminescent reagents [98]. In recent years, researchers have explored the properties of nanomaterials and nanoparticles, which have demonstrated outstanding performance in the detection of early-stage cancer biomarkers.

**Figure 6:**
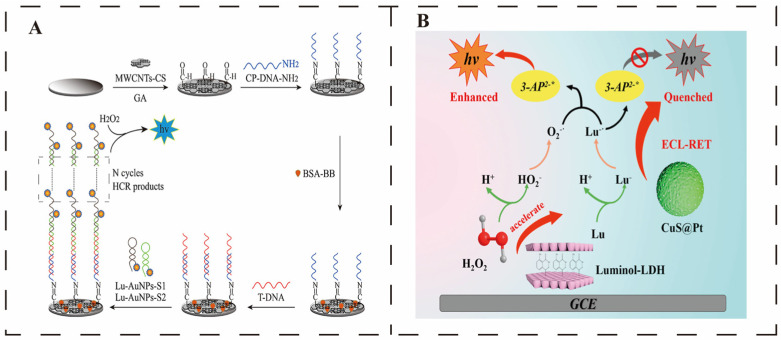
ECL-based nanosensor. (**A**) Composite nanomaterials encapsulate the luminol-modified electrode surface, promoting surface reactions and enhancing sensitivity. ECL signal intensity increases with rising target concentration. Reprinted from [99]. Copyright (2020), with permission from Elsevier. (**B**) ECL-RET sensor employing a dual-potential emitter to amplify ECL signal strength. Reprinted from [102]. Copyright (2023), with permission from Elsevier.

The incorporation of nanomaterials into ECL sensor components can lower the detection limits for tumour markers. As ECL signal intensity increases with target concentration, researchers have explored various nanotechnological strategies to further amplify the output signal. Luminol serves as a classic luminescent moiety. Wu et al. combined luminol–gold nanoparticles with hybrid chain reaction amplification (HCR) to construct a novel fabric-based microfluidic biosensor for detecting p53 [99]. This sensor employs luminol/gold hybrid nanomaterials for signal amplification and probe labelling, while HCR enhances detection sensitivity. A foldable three-dimensional fabric device was fabricated using screen-printed carbon ink and solid wax to create fabric-based carbon electrodes and sample/auxiliary chambers. Leveraging the 3D device’s characteristics, a multi-walled carbon nanotube-modified sensing interface was prepared. Detection was performed by sequentially dispensing the target, signal nanoprobe, and hydrogen peroxide onto the sensing interface (as shown in Figure 6A). This sensor exhibits a detection limit as low as 0.02 fM. Ru(bpy)_3_^2+^ is frequently employed by researchers in ECL sensors, yet its lack of functional groups and high solubility present challenges in application. This issue can be addressed through dual-signal amplification utilising nanomaterials and co-reactive accelerators. Porous, chemically stable P-C_3_N_4_ loaded with CoPdNPs was employed as a reaction accelerator to enhance the ECL signal. Combining sCuO with high-surface-area FeMOFs as a co-reagent to link secondary antibodies further amplifies the response signal. Sequentially modifying both onto an electrode to form a sandwich-type sensor comprises a dual-amplification strategy that enables detection of neuron-specific enolase (NSE) [100]. It must be acknowledged that in ECL reactions, issues such as extended electron conduction pathways, energy dissipation, and the requirement for substantial amounts of co-reactive accelerators cannot be entirely circumvented [101]. Consequently, the exploration of more competitive design approaches is particularly imperative in current research.

Liang and his team constructed a sensor based on the ECL-RET mechanism, which achieves alpha-fetoprotein detection through a self-amplifying system formed by luminol–layered double hydroxide (luminol–LDH) and CuS@Pt [102]. The layered double hydroxide embedded within the luminol acts as a donor, while platinum (Pt) nanoparticles loaded onto the surface of copper sulphide nanospheres function as the acceptor. Through layer-by-layer assembly, the researchers prepared a gold nanoparticle/luminol–LDH composite nanomembrane that not only immobilises luminol but also significantly enhances the detection response signal. ECL-RET quenches luminol, whilst CuS@Pt exhibits visible light capture capability (as shown in Figure 6B). The composite structure formed between the luminescent material and co-reactive species effectively shortens the transport pathway between them, enhancing the efficiency of the ECL reaction. Based on these results, integrating functionalised nanomaterials into ECL sensing systems represents a practical approach for effectively improving detection sensitivity.

Although ECL-based nanosensor strategies continue to be proposed by researchers, their application in clinical diagnostics remains in its infancy. Current key challenges include designing suitable luminescent nanomaterials, broadening their clinical applicability, investigating energy transfer mechanisms in depth, and exploring efficient ECL signal amplification strategies.

### 3.4. Fluorescence-Based Nanoscale Sensors

Fluorescence (FL) represents the most widely employed sensing technology within optical sensors [103], primarily detecting target substances through energy conversion following absorption of short-wavelength radiation. Fluorescent probes comprise materials exhibiting luminescent properties; when these materials interact with target molecules, fluorescent changes occur. Transducers then convert the energy generated during this reaction into detectable signals [104]. Fluorescence sensors are straightforward to design and offer a more cost-effective alternative compared to analytical instruments such as high-performance liquid chromatography and gas chromatography. Building upon the principles of fluorescence-based reactions, researchers have devised multiple sensing strategies. Advancements in nanotechnology and improvements in nanomaterials have significantly impacted sensor development, with the incorporation of nanomaterials into fluorescence sensors playing a crucial role. Nanomaterials can function as fluorescence quenchers, signal probes, and more. Notably, metallic nanoparticles, quantum dots, and carbon-based nanomaterials have demonstrated exceptional optical properties. The rapid development of fluorescence-based nanoscale sensors enables effective identification of tumour markers, aiding clinical diagnosis.

The tumour marker cell cytokeratin fragment 19 (CYFRA 21-1) is a fragment present in numerous normal and malignant epithelial cells, exhibiting markedly elevated levels particularly in the serum of lung cancer patients. Nawal and her team innovatively synthesised carbon quantum dots from lemon peel, combining them with zinc oxide nanomaterials to prepare a carbon quantum dot–zinc oxide nanocomposite [105]. Unlabelled antibodies were immobilised onto the nanocomposite, while another monoclonal antibody was coated onto the surface of a microtiter plate. An immunosensing system was constructed using the sandwich method. Under appropriate excitation and absorption light conditions, the fluorescence intensity was measured to detect CYFRA 21-1 antigen.

Exosomes are vesicular structures secreted by cells that can carry biomacromolecules such as nucleic acids and proteins. Exosomal proteins play a central role within exosomes and are closely associated with tumourigenesis and progression. Liu and colleagues designed a sensor array based on carbon nitride nanosheets modified with aptamers for the fluorescent detection of exosomal proteins [106]. The composite nanoplate structure enhances catalytic efficiency in oxidising o-phenylenediamine (oPD) to the fluorescent molecule DAP. Photoinduced electron transfer (PET) between DAP and carbon nitride nanoplates generates a colourimetric fluorescent signal. In the presence of target molecules, aptamers bind and detach from the nanoplates, reducing catalytic activity and altering the fluorescent signal. Upon addition of organic solvents, only the DAP fluorescence intensity increases, leaving the intrinsic fluorescence of the carbon nitride nanosheets unaffected. This amplifies the aptamer–exosome recognition, lowering the detection limit to 2.5 × 10^3^ per millilitre. This sensing strategy offers novel insights for cancer detection.

To further expand the application of fluorescent sensors, researchers proposed integrating nanomaterials with other systems to enable simultaneous detection of multiple targets. Li et al. proposed an innovative strategy combining an entropy-driven amplification system with a silver nanocluster sensing platform for multiplexed miRNA analysis [107]. This platform integrates target-triggered catalysis with DNA-templated silver nanoclusters exhibiting tunable luminescence. In the presence of targets, the sensing platform undergoes complex migration and chain displacement. The six-base cytosine ring within the complex stabilises the luminescence of silver nanoclusters. Cyclic catalytic reactions of the target molecules can extinguish the fluorescence signal, thereby achieving significant optical signal amplification. In another report, Zhang and colleagues utilised Fe_3_O_4_/TiO_2_ magnetic nanoparticles (MNPs) and CdSe/ZnS quantum dots (QDs) encapsulated within polymer microspheres to fabricate dual-functional magnetic quantum dot barcodes for multiplex tumour marker detection [108]. Fe_3_O_4_/TiO_2_ MNPs mitigate fluorescence quenching in the quantum dot microspheres, enhancing the luminescent effect. This platform enables simultaneous detection of four tumour markers with exceptional sensitivity and accuracy. By designing specific, high-performance nanomaterials, researchers have not only achieved multi-plex detection but also enhanced signal intensity and stability, providing robust support for the early diagnosis and precision treatment of tumour markers.

The quantum effects and enhanced light scattering capabilities of nanomaterials offer unique properties for designing highly sensitive sensors, demonstrating significant potential for fluorescence enhancement. Although fluorescence sensing technology has made considerable progress, shortcomings persist, such as cumbersome and complex operation procedures and time-consuming processes. Subsequent research may enhance the luminescence efficiency and signal stability of fluorescent probes through morphological adjustments to nanomaterials and the preparation of sensor arrays, thereby enabling efficient detection of tumour markers.

In the fields of early cancer screening and human health monitoring, electrochemical and optical sensing technologies have demonstrated numerous advantages (see Table 2). Comparing the two sensor types, several key trends emerge: 1. Optimal detection limit strategy—‘MOF@metal nanoclusters + cascade reactions’ generally achieve lower detection limits through dual amplification of signals. However, as most tumour markers’ clinical diagnostic ranges fall within ng/mL to μg/mL, femtomolar (fM) detection limits already meet early diagnosis requirements. Optimising the minimum detection threshold must align with clinical needs. 2. Optimal stability strategy—‘Carbon-based materials (graphene/rGO) + metal nanoparticles’ (e.g., rGO-AuPt) exhibit minimal batch-to-batch variation in detection limits, whereas optical sensors’ stability relies on metal nanostructures. 3. Optimal linear range strategy—Electrochemical sensors possess broader linear ranges than optical biosensors, better accommodating target concentration fluctuations in clinical samples. 4. Optimal Detection Signal Strategy—Optical sensors support multi-signal integration, reducing interference and enhancing accuracy. 5. Suitability of clinical samples—Current research predominantly involves small-scale testing, with most samples being serum dilutions or cell lysates. ‘Anti-contamination modification of nanomaterials’ is pivotal for clinical application.

## 4. Clinical Applicability Assessment

Researchers continue to develop high-performance sensors with the ultimate aim of achieving practical clinical applications. After reviewing advances in nanomaterial-mediated electrochemical and optical sensors for tumour marker detection, we assessed their suitability for clinical implementation. We consulted clinical trial databases and summarised recent cases of sensor application in clinical cancer diagnosis, as shown in Table 3.

Transitioning nanomaterial-mediated electrochemical and optical biosensors from laboratory research to clinical application presents multiple challenges encompassing multi-faceted factors. (1) Cost analysis: The relatively high price of individual nanomaterials necessitates cost reduction through scaled-up production. (2) Mass production: Laboratory preparation often relies on manual operations, where nanomaterial synthesis is prone to variations that impact detection limits. (3) Sensitivity versus practical application: Complex mechanisms within clinical samples may induce interference, leading to false negative or false positive results. (4) Complexity of clinical validation: Verifying consistency across diverse patient cohorts demands substantial time and funding, often beyond the capacity of typical laboratories. (5) Sensor detection times of approximately 30–60 min remain insufficient for emergency ‘rapid diagnosis’ protocols. (6) Regulatory approvals remain pending, with only partial completion of clinical trials to date and no authorisation for use. Clinical translation case studies indicate that electrochemical sensors employing ‘carbon-based materials + electrodeposition techniques’ offer low-cost, scalable production with high sensitivity, demonstrating clinical implementation potential. Liu’s team developed a distributed-fluorescence-enhanced metasurface nanoplasmonic (DFE-MSPR) chip POCT device, which has been approved for commercialisation. This enables sensitive detection of small molecules in blood, outperforming ELISA in both sensitivity and speed [109]. This provides a viable pathway for the clinical application of nanobiosensors.

## 5. Future Outlook

In future developments, the paramount priority lies in researching more rapid, sensitive, cost-effective, and high-performance nanosensing strategies. We must not only deepen our understanding of the operational mechanisms of various sensors but also enhance our comprehension of cancer heterogeneity, effectively integrating both aspects [110]. Addressing current challenges, future research should focus on the following directions: 1. Development of biocompatible nanomaterials. For instance, creating polysaccharide-loaded nanoenzymes or designing framework systems to quantify the biological response thresholds of nanomaterials to the body. By optimising the specific surface area and morphology of nanomaterials to reduce their toxicity, negative inflammation can be decreased by 36.19% [111]. 2. Maintaining uniform precision in the large-scale production of nanomaterials. Scaled manufacturing reduces batch-to-batch variability while lowering production costs. 3. Integration of diverse technologies. With the rapid advancement of intelligent systems, incorporating machine learning algorithms optimises sensor signal analysis. When processing vast datasets, these algorithms extract the most pertinent or valuable aspects, enabling effective analysis and enhancing detection capabilities [112]. Flexible nanomaterials can be utilised to fabricate wearable sensors, enabling real-time daily monitoring through smartphone connectivity. 4. Optimising clinical translation pathways: Enhanced collaboration with clinicians is essential, using actual clinical needs as the starting point to design sensors that better meet requirements. 5. Multiplexed detection represents a key direction. Developing a ‘sensor array + multi-biomarker combination’ model can incorporate emerging nanobodies, enabling simultaneous detection of multiple biomarkers for the same cancer using a sensor array, thereby improving early cancer diagnosis rates. Advances in nanotechnology drive the design of nanomaterials and their integration with sensors, contributing to progress in cancer diagnostics

## 6. Conclusions

Early detection of cancer facilitates simpler treatment, reduces therapeutic costs, and improves patient survival rates. Consequently, early screening for various cancers can be considered the most crucial step in treatment protocols. This review summarises recent research on nanomaterial-mediated electrochemical and optical biosensors, drawing the following conclusions: ① The signal amplification mechanisms of nanomaterials (enzyme-like activity, interfacial optimisation, high specific surface area) are central to enhancing sensor detection performance. The dual amplification strategy of “MOF@metal nanoclusters + cascade reactions” demonstrates advantages in achieving low limits of detection (LOD). ② Electrochemical sensors better meet the requirements of point-of-care testing (POCT), offering lower production costs and ease of miniaturisation. Optical biosensors support detection across multiple sample types, facilitating deeper investigation into tumour marker origins and mechanisms of action. ③ Emerging nanobody materials (VHH, VNARs, and VLRs) exhibit unique advantages in sensor construction due to their small molecular weight and tolerance to high temperatures and salinity, enabling detection in harsh environments. ④ Key challenges for clinical translation include bottlenecks in large-scale production and regulatory approval processes.

By comparing the detection performance and clinical applicability of electrochemical and optical biosensors, this study offers potential pathways for the clinical translation of nanomaterial-mediated biosensors. However, existing research remains largely confined to in vitro validation, necessitating further multi-centre, large-scale studies for verification. Strengthening the integration of research with clinical needs will facilitate the advancement of nanosensors into cancer diagnosis clinical practice.

## Figures and Tables

**Table 2:** Comparative Analysis of Comprehensive Performance in Nanomaterial-Mediated Biosensors.

Classification	Nanosensing Strategy	Target	Linear Range/ LOD	Sample Type	Research and Development Phase	Ref.
Electrochemical Biosensor	Nanoenzymes catalyse substrate reactions on electrode surfaces, enhancing detection signals.	miRNA let-7a	0.4–140 nM/ 0.25 nM	Hela cells	In vitro	[43]
	The marker–target–probe combination achieves signal amplification.	miRNA let-7a miRNA-21	0.01–10 pM/3.6 fM 0.02–10 pM/8.2 fM	Human serum	Small-sample validation	[71]
	Nanozymes and the Cascade Primer Exchange Reaction (PER).	miRNA-21	1 fM–1 nM/ 0.29 fM	Human serum	Small-sample validation	[44]
	Carboxylated graphene oxide modifies the electrode surface, with gold nanoparticles mediating target recognition.	miRNA-21	1 fM–1 μM/ 1 fM	Human serum	Small-sample validation	[56]
	Metal ion functionalisation for preparing composite sensing interfaces enabling simultaneous detection of multiple biomarkers.	miRNA-21 miRNA-155 miRNA-210	0.001–1000 pM/ 0.04 fM 0.33 fM 0.28 fM	Human serum	Small-sample validation	[59]
	Functionalised reduced graphene oxide probes react with electroactive molecules modified onto electrodes, yielding an oxidation peak.	miRNA-21	8 fM–2.0 pM/ 5.4 fM	Human serum	Small-sample validation	[67]
	Nanozyme-catalysed in situ deposition of silver nanoparticles enhances the response signal.	GPC3	0.01–10 μg/mL /3.30 ng/mL	Human serum	Small-sample validation	[48]
	Nanoenzymes and natural enzymes jointly catalyse the reaction.	AFP	0.02–100,000 pg/mL /0.01 pg/mL	Human serum	Small-sample validation	[45]
	Graphene provides binding sites for metallic nanoparticles, enhancing the response signal.	H_2_O_2_	0.01–0.05 × 10^−6^ M 0.15–8623 × 10^−6^ M /2.8 × 10^–9^ M	Mouse	In vitro	[68]
	Nano-composites with enhanced electrocatalytic performance, optimised sensing interface	CA72-4	0.001~500 U/mL /1.78 × 10^−5^ U/mL	Human serum	Small-sample validation	[55]
	Three-dimensional nanocrystals have been grafted with functional groups to enhance detection sensitivity.	CEA	0.001–100 ng/mL /0.23 pg/mL	Human serum	Small-sample validation	[70]
Optical biosensor	Surface-enhanced Raman scattering enhanced by metallic elements	CYPA	-/7.76 × 10^−10^ μg/mL	Human plasma	Small-sample validation	[84]
	Surface-enhanced Raman scattering and fluorescence dual-signal detection.	MUC1	-/1.16 fg/mL 1.19 fg/mL	Cancer cells	In vitro	[85]
	Reaction between nanomaterials and target substances, followed by structural alteration to achieve signal amplification.	CD63	55–5.5 × 10^5^ particles/μL /17 particles/μL	Cancer cells	In vitro	[87]
	Nanozyme catalysis, smartphone imaging.	ALP	0.001–100 U/L /0.47 mU/L	Human serum	Small-sample validation	[91]
	The LSPR of metallic particles interacts with antibodies, incorporating silver nanoparticles to optimise detection ratios.	HER	0.5–40 × 10^−7^ M /3.7 × 10^−9^ M	Human serum	Small-sample validation	[94]
	LSPR-enhanced Ag@Au nanostructure biosensor	miRNA let-7a	0.1 pM–10 aM /5.45 aM	Human serum	Small-sample validation	[95]
	ECL-RET sensor with dual-emitter system.	AFP	10^−5^–100 ng/mL /2.6 fg/mL	Human serum	Small-sample validation	[102]
	Reaction accelerator composite nanomaterial P-C_3_N_4_-CoPd NPs modifies electrode, promoting conversion of K_2_S_2_O_8_ into active intermediate of luminescent system.	NSE	0.00005–100 ng/mL /20.4 fg/mL	Human serum	Small-sample validation	[100]
	Construction of a Fluorescent Sensing System Based on the Sandwich Method.	CYFRA 21-1	0.01–100 ng/mL /0.008 ng/mL	Human serum	Small-sample validation	[105]
	PET-effect-based fluorescent colorimetric sensing platform.	Exosomes	-/2.5 × 10^3^ per mL	Human serum	Small-sample validation	[106]
	Combining an entropy-driven amplification system with silver nanoclusters for dual-signal amplification.	miRNA-141 miRNA-155	-/6.1 pM 8.7 pM	Tumour tissue	In vitro	[107]

**Table 3:** Classification of stages and clinically feasible technologies for sensors in cancer diagnosis.

Sensor Type	Method	Clinical Trial	Cancer Type	Clinical Standard	Classification	Year
Electrochemical Sensor	Impedance Method	NCT03929185	Various Types	Pathological Diagnosis	Stage Technology/ Early trial	2019
	-	NCT04825002	Prostate Cancer	Pathological Diagnosis	Stage Technology/ Early trial	2021
	Impedance Method	ChiCTR2200058608	Endometrial Cancer	Pathological Diagnosis	Early trial	2022
Optical Sensor	SERS	NCT06772376	Non-Small Cell Lung Cancer	Pathological Diagnosis	Stage Technology/ Early trial	2024
	Optical spectromete	NCT02943044	Head and Neck Squamous Cell Carcinoma	Pathological Diagnosis	Stage Technology/ Early trial	2017
	-	NCT02068378	Lung Cancer	Pathological Diagnosis	Completed trial	2018
	FL	NCT02957370	Bladder Cancer	cystoscopy	Stage Technology/ Early trial	2021
Semiconductor Sensor	Impedance Method	NCT06211010	Tumors of the urinary system	Pathological Diagnosis	Completed trial	2024
Nanopore Sensor	Nanopore	ChiCTR1900023951	Breast Cancer	Pathological Diagnosis	Early trial	2019

## Data Availability

The data used in this review are derived from public resources, including but not limited to well-known academic databases, publicly available government reports, and authoritative websites in specialized fields. The acquisition of all data complies with relevant laws and regulations, as well as the terms of use stipulated by the data providers. For data obtained from academic databases, their sources have been detailed in the references, and readers can trace the original data based on the cited information. Data from government reports can be accessed through the official websites of the respective government departments. Data from authoritative websites in specialized fields are provided with links or clear source descriptions at appropriate locations in the text. The purpose of this statement is to ensure the transparency and reproducibility of the data, thereby promoting the healthy development of academic research. If you have any special requirements regarding the sources and acquisition methods of the data in the report, or if you would like to provide additional information, please feel free to let me know.
